# Iridoschisis: une condition rare

**DOI:** 10.11604/pamj.2024.49.135.45888

**Published:** 2024-12-27

**Authors:** Malek Kharrat, Yosra Maalej

**Affiliations:** 1 Ophthalmology Department, Mohamed Taher Maamouri Hospital, Faculty of Medicine Tunis El Manar, Tunis, Tunisia

**Keywords:** Iridoschisis, hypertonie, glaucome par fermeture de l’angle, Iridoschisis, epithelium, stroma

## Abstract

Iridoschisis, a rare degenerative condition, is defined by atrophy of the iris associated with cleavage of the iris stroma, the anterior part of which disintegrates into fibrils floating in the Anterior chamber (AC). Iris involvement is bilateral, progressive but not transfixing, respecting the pigment epithelium and often localised in the lower sector. Diagnosis is clinical. Optical coherence tomography of the anterior segment (OCT-SA) provides characteristic semiological features. The main differential diagnoses are irido-corneo-endothelial syndrome and Axenfeld-Rieger syndrome. Association with angle-closure glaucoma (ACG) is reported in 2/3 of cases. The mechanism of hypertonia is thought to be twofold: failure of the trabecular meshwork to evacuate Aqueous humour (AH) through iris debris and resistance of the iridolenticular interface to the flow of AH, resulting in pupillary block. A 71-year-old patient with no previous pathological history consulted for a red and painful left eye in connection with an acute angle-closure crisis. After resolution of the crisis by hypotonising treatment, examination showed a narrow CA (A), an atrophic iris inferiorly cleaved into fibrils floating in the HA (B). Gonioscopy showed a closed Iridocorneal angle (ICA) in the absence of anterior synechiae. OCT-SA showed cleavage and disorganisation with a mottled appearance of the anterior part of the iris stroma respecting the iris pigment epithelium (C), a closed ICA and a narrow CA (D). All these features supported the diagnosis of iridoschisis associated with an AFM crisis.

## Image en médecine

L'iridoschisis, une pathologie dégénérative rare, se définit par une atrophie de l'iris associée à un clivage au sein du stroma irien dont la partie antérieure se désintègre en fibrilles flottant dans la chambre antérieure (CA). L'atteinte est bilatérale, évolutive mais non transfixiante de l'iris respectant l'épithélium pigmentaire, souvent localisée dans le secteur inférieur. Le diagnostic est clinique. La tomographie par cohérence optique du segment antérieur (OCT-SA) apporte des éléments sémiologiques caractéristiques. Les principaux diagnostics différentiels sont le syndrome irido-cornéo-endothelial et le syndrome d'Axenfeld-Rieger. L'association avec un glaucome par fermeture de l'angle (GFA) est rapportée dans 2/3 des cas. Le mécanisme de l'hypertonie serait double: défaut d'évacuation de l'humeur aqueuse (HA) par les débris de l'iris dans le trabéculum et la résistance de l'interface irido-lenticulaire au flux d'HA responsable d'un blocage pupillaire. Un patient âgé de 71 ans sans antécédent pathologique ayant consulté pour un œil gauche rouge et douloureux en rapport avec une crise aiguë par fermeture de l'angle. Après résolution de la crise par un traitement hypotonisant, l'examen montrait une CA étroite (A), un iris atrophique en inférieur clivé en fibrilles flottant dans l'HA (B). La gonioscopie a objectivé un angle iridocornéen (AIC) fermé en l'absence de synéchies antérieures. L'OCT-SA a montré un clivage et une désorganisation avec un aspect mité de la partie antérieure du stroma irien respectant l'épithélium pigmentaire de l'iris (C) un AIC fermé, et une CA étroite (D). Tous ces éléments plaidaient en faveur du diagnostic de l'iridoschisis associé à une crise de GFA.

**Figure 1:**
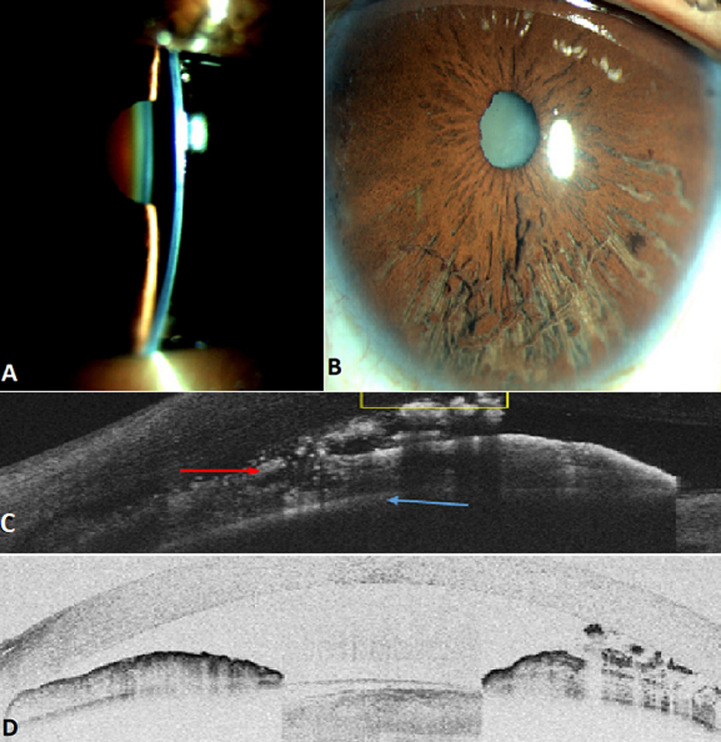
segment antérieur de l'iris avec des coupes OCT de l'œil gauche: (A) CA étroite; (B) iris atrophique en inférieur clivé en fibrilles flottant dans l'HA; (C,D) clivage, désorganisation et un aspect mité de la partie antérieure du stroma irien respectant l'épithélium pigmentaire de l'iris avec un AIC fermé, et une CA étroite

